# Socioeconomic Status, the Home Language Environment, Noise Exposure, and the Mismatch Response in Infancy

**DOI:** 10.1002/dev.70128

**Published:** 2026-03

**Authors:** Katrina R. Simon, Belen Azofra Macarron, Aislinn Sandre, Melina Amarante, Sonya V. Troller-Renfree, Kimberly G. Noble

**Affiliations:** 1 Mailman School of Public Health, Columbia University, New York City, New York, USA; 2 Universite Paris Cité, Paris, France; 3 University of Western Ontario, London, Ontario, Canada; 4 Fordham University, New York City, New York, USA; 5 Teachers College, Columbia University, New York City, New York, USA

## Abstract

Socioeconomic resources have long been associated with children’s language development. Several proximal factors have been suggested as candidate mechanisms underlying socioeconomic disparities in language development, including differences in the home language environment and home noise levels. These experiences may in part shape auditory discrimination skills, a key component of language comprehension. To index early auditory discrimination, researchers measured brain function in relation to the detection of different sounds with an event-related potential (ERP) called the mismatch response (MMR). The current study aimed to examine associations among socioeconomic circumstances, the home language environment, home noise exposure, and the MMR in a socioeconomically, racially, and ethnically diverse longitudinal sample of 6- and 12-month-old infants. Socioeconomic circumstances were measured prenatally via parent report. The home language environment and home noise levels were measured using digital language processing devices when infants were approximately 6 months of age. The MMR was elicited during a passive auditory oddball task at two timepoints—6 and 12 months of age. Results showed that neither SES, the home language environment, nor home noise levels predicted infant MMR at either age. These findings add to a growing body of literature examining the role of distal and proximal factors in shaping infant brain activity related to language development.

## 1 | Introduction

Family socioeconomic status (SES), typically indexed by family income-to-needs (ITN) ratios, parent education level, and/or ratings of subjective social status, is consistently associated with disparities in language outcomes (Fernald et al. 2013; Lee and Burkam 2002; Noble et al. 2015). Such disparities may emerge early in indices of neural processing, with SES shaping infant responses to speech and environmental input (Tomalski and Johnson 2010; Tomalski et al. 2013; Stevens et al. 2009). These associations are likely driven by more proximal factors within children’s environments, such as characteristics of the home language environment (Gilkerson et al. 2018; Caskey et al. 2014; Rowe et al. 2012; Zimmerman et al. 2009) and noise exposure levels (Maxwell and Evans 2000; White-Schwoch et al. 2015).

Children from families with greater socioeconomic resources generally are exposed to home language environments with more adult words and more frequent conversational turns (Hart and Risley 1995; Rowe et al. 2012; Merz et al. 2020). These characteristics of the home language environment are linked to both language outcomes (Rowe et al. 2012) and neural structure and function that support language development (Pace et al. 2017; Romeo et al. 2018, 2018, 2022; Merz et al. 2020). In contrast, children from families with fewer socioeconomic resources are more likely to experience fewer opportunities for parent–child interaction or learning experiences (Schwab and Lew-Williams 2016) and higher environmental noise levels (Casey et al. 2017), all of which have been associated with worse language development (Perkins et al. 2013; Schwab and Lew-Williams 2016; Erickson and Newman 2017). Consistent with the broader literature showing that early neurocognitive mechanisms contribute to later language (Riva et al. 2017; Kuhl 2011), everyday language exposure may strengthen infant auditory encoding and discrimination, which in turn may shape neural indices that support early language development.

Notably, it has been theorized that higher levels of environmental noise, defined as unwanted or unattended sound (Erickson and Newman 2017), may also interfere with auditory processing abilities (Newman 2009; Evans and Kantrowitz 2002). This might be particularly detrimental for infants, as higher home noise levels may render fewer opportunities to clearly hear and engage in linguistic exchanges that would contribute to the development of auditory processing. Increased exposure to noise has been shown to degrade auditory discrimination in children (Niemitalo-Haapola et al. 2015) and has been linked to characteristics of brain regions that support language development and processing (Simon et al. 2022). However, no studies have examined whether naturally occurring home noise levels relate to infants’ auditory discrimination abilities.

Auditory discrimination—the ability to detect subtle differences in speech sounds—is an essential component of language development (Kujala 2007). At the neural level, auditory discrimination can be assessed using event-related potentials (ERPs) such as the mismatch response (MMR). The MMR is commonly elicited using an auditory oddball paradigm where individuals hear a series of frequent (“standard”) and infrequent (“deviant”) sounds that differ slightly in specific acoustic features (e.g., frequency, pitch). Importantly, the standard and deviant sounds typically vary only slightly by acoustic feature, allowing the difference in neural response to the deviant versus the standard (i.e., the MMR) to reflect the neural ability to detect small deviations in physical characteristics, thus indexing auditory discrimination (Kujala and Naatanen 2010). In older children, adolescents, and adults, the MMR often appears as a negative deflection and is commonly referred to as the mismatch negativity (MMN). However, in infancy and early childhood, this response typically shows a positive deflection and is thus referred to as the MMR (Benasich et al. 2006; Choudhury and Benasich 2011; Garcia-Sierra et al. 2016). Because the MMR indexes automatic detection of subtle acoustic changes, it is well suited for capturing experience-based differences arising from variation in early language and noise exposure.

The MMR is thought to reflect automatic auditory change-detection processes generated primarily in the auditory cortex and frontal regions (Näätänen et al. 2007; Garrido et al. 2009). In adults, the response occurs from the brain’s predictive coding mechanisms, in which the auditory system forms expectations based on expected sound patterns and produces an MMR when a deviation violates expectations. In infants, the positive MMR is believed to reflect an earlier form of this mechanism, potentially reflecting maturational differences in cortical organization, synaptic density, and excitation-inhibition balance within the auditory cortex (He et al. 2009; Kushnerenko et al. 2002). As such, although polarity of the MMR differs across development, both responses index the neural ability to discriminate and encode changes in auditory input, processes that support early language learning (Cheng et al. 2015; Friederici et al. 2007; Kujala 2007; McWeeny and Norton 2020). Variations in children’s environments, such as differences in language input, have been associated with differences in MMR amplitude and latency (Garcia-Sierra et al. 2016; Marklund et al. 2019). Further, MMR amplitude in children has been associated with both concurrent (Holopainen et al. 1997; Grossheinrich et al. 2010; Norton et al. 2021) and subsequent (Choudhury and Benasich 2011; Schaadt et al. 2015; Fan et al. 2023) language skills in children (see Kujala and Leminen 2017 for a review). Similarly, evidence suggests that early rapid auditory processing abilities in infancy may mediate genetic influences on expressive language development (Riva et al. 2018), underscoring the role of early auditory neural processing in shaping language outcomes.

Links between language exposure and MMR amplitude have been observed, with infants exposed to more speech displaying larger (i.e., more positive) MMR amplitudes (Garcia-Sierra et al. 2016; Marklund et al. 2019). Past work has also observed smaller (i.e., less positive) MMRs when elicited in noisy environments in adults (Kozou et al. 2005; Koerner et al. 2016) and children (Niemitalo-Haapola et al. 2017), but no work has examined how the MMR might differ in relation to variations in ambient everyday noise exposure. Socioeconomic circumstances have been linked to differences in the MMR, though evidence is mixed, with some studies reporting that lower subjective social status in young adults was associated with more negative MMRs (Hao and Hu 2024), and others finding no relationships between SES and the MMR in school-age children (Anwyl-Irvine et al. 2021). Importantly, no studies have examined associations between SES and the MMR in infancy. Similarly, no work has examined how SES and everyday experiences, including conversational turn counts (CTCs), adult word counts (AWCs), and ambient noise levels, might shape infant auditory processing. Further, it remains unknown as to whether the home language environment and noise exposure might mediate associations between SES and early auditory discrimination as reflected by the MMR. Examining whether these factors predict early auditory discrimination abilities can contribute to a better understanding of how early experiences shape neural function related to later language development.

As such, the current longitudinal study aimed to examine associations among SES, the home language environment, home noise levels, and the MMR during two time points in infancy. We hypothesized that (1) infants from lower SES homes, (2) those engaged in fewer conversational turns, and (3) those exposed to fewer adult words would all exhibit smaller (less positive) MMRs. We also hypothesized that infants from homes with higher home noise levels would exhibit smaller (less positive) MMRs. Finally, we hypothesized that the home language environment (AWCs and CTCs) and home noise levels would mediate associations between socioeconomic circumstances and the MMR.

## 2 | Methods

### 2.1 | Participants

Mothers were recruited from local prenatal clinics, community events, and social media to participate in a longitudinal study on child development. Participants were from the New York City metropolitan area and were recruited to span a range of educational attainment, from having less than a high school education to holding an advanced degree. Mothers were recruited over two time periods because of a temporary interruption in data collection due to the COVID-19 pandemic. A total of 209 mothers were enrolled in the longitudinal study after completing the prenatal visit, with 93 recruited between June 2019 and March 2020 (Cohort 1) and 116 recruited between August 2021 and September 2022 (Cohort 2). To participate, mothers were required to be 18 years of age or older, speak English or Spanish, be carrying a singleton fetus with no known neurological or developmental issues, and be at least 35 weeks pregnant. Once eligibility was confirmed, mothers were invited to participate in a prenatal visit in the lab or their home.

After the birth of the infant, eligibility for successive study visits was confirmed for subsequent participation. Inclusion criteria for infants included: Gestational age greater than or equal to 37 weeks and no known neurodevelopmental issues at birth. Mothers were contacted every six months for follow-up visits. One mother–infant dyad was excluded from the study due to birth-related complications, and five mothers unenrolled from the study by the time of contact for the 6-month visit. Two-hundred and three mother-infant dyads were subsequently invited to complete a lab or home visit when the infant was approximately 6 and 12 months of age. Of these participants, 189 mother-infant dyads completed the six-month visit, virtually or in-person (Cohort 1: *n* = 84; Cohort 2: *n* = 105) and 178 dyads completed the 12-month visit, either virtually or in-person (Cohort 1: *n* = 75; Cohort 2: *n* = 103). Due to restrictions on in-person data collection related to the COVID-19 pandemic, EEG collection was only possible for infants in cohort 2.

### 2.2 | Measures

#### 2.2.1 | Family SES

At the prenatal time point, mothers completed a questionnaire that assessed educational attainment (total years of education for mother and father separately), household composition (number of adults and children in household), and family income (estimated gross annual income). Parent educational attainment was calculated by averaging the number of years of education attained by the mother and the second parent. In cases where the reporting mother was the sole parental caregiver, only maternal educational attainment was used. Parent educational attainment for at least one parent was reported by all 209 mothers. Seventeen mothers selected “prefer not to answer” for the family income question; therefore, income data were available for 192 mother–infant dyads (i.e., 92% of the full sample). Family ITN ratios were calculated by dividing participants’ total household income by the U.S. poverty threshold for the respective family size for the year of data collection. An ITN below 1 indicates that a family is living below the federal poverty threshold, whereas an ITN above 1 indicates that a family is living above the federal poverty threshold. Fifty-two mothers (i.e., approximately 27% of the sample who provided income data) reported an ITN of below the poverty line. As expected, ITN values were positively skewed and subsequently log-transformed for use in all analyses. Fourteen participants reported that their income was zero dollars. To enable log transformation, one dollar was added to all income values prior to calculating ITN to account for reported family income of zero dollars. Three ITN values were detected as outliers (defined as values greater than 3 standard deviations above the mean), and as such results with ITN as the independent variable of interest were run with both winsorized and non-winsorized values. We report analyses using winsorized values below; (see Table S2 for results using non-winsorized ITN values.

#### 2.2.2 | Infant Demographic Factors

Infant demographic factors, including assigned sex, race, and ethnicity were collected from mothers via questionnaire at the 1-month time point. Thirteen mothers selected “prefer not answer” on the item that assessed infants’ race, and 5 mothers selected “prefer not to answer” on the item that assessed infants’ ethnicity. Further, one parent selected “prefer not to answer” when selecting infant assigned sex at birth.

#### 2.2.3 | Home Language Environment

The Language ENvironment Analysis (LENA) digital language processor (LENA Research Foundation, Boulder, CO) was used to measure the home language environment. LENA is an automated vocalization analysis device that can audio-record the child’s language environment for up to 16 hours. At the 6-month visit, participants who agreed to take part in the LENA activity were given the device along with two infant t-shirts and instructions on use (i.e., mothers were instructed to have the infants wear the t-shirt with the LENA device turned on inside the pocket for a whole day, removing it when the baby was napping for comfort). The research team also advised mothers to complete this activity on a day when they spend most of the time with their infant (e.g., a weekend day).

Once returned, audio recordings from the device were downloaded and analyzed using LENA software. This software provides several automated measures including—the number of words a child hears from an adult (AWC); the number of conversational turns (i.e., a child utterance followed by an adult utterance within 5 s, or vice versa; referred to as the CTC); the number of child vocalizations (i.e., child’s speech of any length surrounded by > 300 ms of silence or any other sound not produced by the child (child vocalization count or CVC); the number of seconds the child is exposed to electronic media (i.e., TV, iPad); and background noise. High validity and reliability of the LENA speech identification algorithms have been reported for adult and infant speech (Gilkerson et al. 2017), both in English and in Spanish (Weisleder and Fernald 2013). Relevant to the current study, hourly AWC, hourly CTC, and noise levels were extracted from the LENA.

Further data processing took place to remove silent periods of 10 minutes or more from recordings to ensure that LENA analyses were limited to times in which the infant was awake. To remove these silent periods, custom Python scripts (see github.com/trollerrenfr/LENA_Scripts) were used. Segments were removed from the data when they consisted of two consecutive 5-min periods in which the LENA automatic algorithm detected one or more of the following: (1) There was noise or silence for more than 180 seconds; (2) there were no adult words detected; (3) there were no conversational turns detected; and (4) there were fewer than 10 child vocalizations. With the remaining data, LENA counts were divided by the duration of the LENA recording, to obtain average hourly counts of adult words and conversational turns. Values were queried for outliers, with one hourly CTC identified as an outlier (> 3 standard deviations from the mean). As such, this value was winsorized, and analyses using CTC were run with both the original value and the winsorized value. Of the 137 participants who consented to participate in the LENA activity, 11 participants either lost the device or returned it without data. Recordings shorter than 5 hours (*n* = 4) were excluded from subsequent analyses, leaving a sample size of 122 with usable metrics for the home language environment and noise. Upon closer inspection of the raw data, 5 participants exhibited device usage that spanned the course of multiple days (>2 days) or repeated instances of turning the device on/off (>3 pauses of the device). As such, we ran robustness checks in which analyses using LENA variables as a predictor were rerun with and without these data.

#### 2.2.4 | Home Noise Levels

To calculate average home noise levels, LENA recording files for each participant were entered into the LENA Advanced Data Extractor (ADEX; Xu et al. 2008). Using the ADEX tool, LENA data were uploaded and average sound pressure level in decibels were automatically extracted for each participant. One participant exhibited noise levels greater than 3 standard deviations from the mean; this value was winsorized.

#### 2.2.5 | Child Bilingualism Status

Bilingualism status was assessed at the 6-month time point via maternal report. Parents were asked what language was mainly spoken in the home, followed by a question regarding how often that language was spoken. Possible answers were “Mostly”, “Almost Always”, and “About half the time”. Mothers were also asked whether children were exposed to any other languages. Answers to this question were then dummy coded, with a “1” coding any level of bilingualism, and a “0” coding no exposure to another language in the home. In our full sample of 209 infants, 52 infants were classified as bilingual and 134 infants were classified as monolingual, with 23 infants missing bilingualism information.

#### 2.2.6 | ERP Task (Cohort 2 Only)

EEG collection occurred at both the 6- and 12-month time points for infants who attended an in-person visit. Prior to ERP data collection, infants participated in a 3-min baseline EEG task to measure neural activity at rest. During ERP acquisition, infants were held in their mothers’ arms in a dimmed room while completing a 2-stimulus auditory oddball paradigm (a frequent, standard stimulus and infrequent, deviant stimulus) to elicit an MMR. Stimuli were computer-generated at 48 kHz, 16-bit resolution consonant-vowel syllables /ba/ and /da/. Stimuli were presented using a task structure of 666 stimuli (566 or 85% standard stimuli; 100 or 15% deviant stimuli), following task structures of Dr. April Benasich (Rutgers University Newark). Speech sounds were selected to elicit MMR due to prior work suggesting that the MMR in response to speech sounds are more closely to later language outcomes compared to the MMR elicited by tonal stimuli (Fox et al. 2024; Norton et al. 2021; Alonso-Búa et al. 2006; Neuhoff et al. 2012). This specific stimulus contrast was selected as it represents an acoustically subtle yet meaningful contrast in both the English and Spanish, the two dominant languages heard by infants in our sample. This contrast has been widely used in infant ERP studies and has been shown to reliability elicit an MMR (Uhler et al. 2021). Stimuli were presented in a sound-attenuated environment using E-Prime software (Psychology Software Tools, 2016). Each stimulus was presented for 100 ms at 70 dB SPL and the interval between sound onsets was 1020 ms, following the specifications of similar tasks (Ortiz-Mantilla et al. 2019). All deviant stimuli were preceded by at least 3 and at most 10 standard stimuli, and stimuli were presented in the same sequence for each infant. A soundless infant video played in the background for children to attune to visually. Mothers were instructed by research assistants to talk and interact with their infant as little as possible during ERP data collection.

#### 2.2.7 | Electroencephalography Data Acquisition and Processing (Cohort 2 Only)

EEG was recorded using a 128-channel montage HydroCel Geodesic Sensor Net (Magstim Electrical Geodesic Inc., Eugene, OR, USA) and on a Net Amps 400 amplifier. Whenever possible, all electrode impedances were kept below 50 kΩ. The sampling rate was 1000 Hz and data were online referenced to the vertex (Cz) electrode.

Offline processing was completed using the EEGLAB toolbox (version 2023.1; Delorme and Makeig 2004), MATLAB (Version R2021b; The MathWorks, Natick, MA), and the Maryland analysis for Developmental EEG (MADE) pipeline (Debnath et al. 2020). Prior to filtering, the outermost ring of electrodes (i.e., electrodes located near the base of the skull: 17, 38, 43, 44, 48, 49, 113, 114, 119, 120, 121, 125, 126, 127, 128, 56, 63, 68, 73, 81, 88, 94, 99, 107) were removed because these electrodes tend to have poor connections and are highly susceptible to noise in infant samples (Debnath et al. 2020). Data were then high-pass filtered at 1 Hz and low-pass filtered at 15 Hz, following previous developmental work (Kushnerenko et al. 2007; Mussacchia et al. 2017). A 1 Hz high-pass cutoff is common in developmental EEG to reduce drift and movement artifacts, though it may attenuate slower ERP components such as the MMR (Luck 2014; Widmann and Schröger 2012). Bad channels were identified and removed using the EEGLAB plug-in FASTER (Nolan et al. 2010). Ocular artifacts and generic noise were removed by creating a copy of the dataset and performing independent component analysis (ICA) on the copied data. The copied dataset was high pass filtered at 1 Hz and segmented into arbitrary 1000 ms epochs. Epochs were removed from this copied dataset if the amplitude was ±1000 μV or if power in the 20–40 Hz band (after Fourier analysis) was greater than 30 dB (Brito et al. 2020; Troller-Renfree et al. 2020). If more than 20% of the epochs in a given channel were removed, that channel was excluded from both the ICA-copied dataset and the original dataset (Debnath et al. 2020; Troller-Renfree et al. 2020). Following this, ICA (Comon 1994; Makeig et al. 1997) was performed on the copied dataset and the ICA weights were copied back to the original continuous data (high pass filtered at 1 Hz). The adjusted-ADJUST toolbox (Leach et al. 2020) was used to automatically identify artifactual independent components (ICs) in the original dataset. All artifactual ICs were removed from the data. Next, data were epoched into 900 ms segments that started 100 ms before sound onset.

After ICA artifact removal and epoching, a two-step procedure for identifying residual artifacts was used. First, to ensure ocular artifacts were removed, epochs where any of the frontal electrodes (i.e., 1, 8, 14, 21, 25, 32) exceeded a voltage threshold of ±150 μV were rejected from all further analyses. Second, for any epoch in which non-ocular channel voltages exceeded ± 150 μV, we interpolated these channels at the epoch level. However, if more than 10% of channels exceeded ±150 μV, we rejected the epoch. In addition, if all epochs were deemed artifactual for an infant (i.e., more than 10% of channels were interpolated on every epoch), that infant was excluded from subsequent processing (ERP data at 6-months removed *n* = 1; ERP data at 12 months removed *n* = 2). Finally, we interpolated all missing channels using a spherical spline interpolation and then referenced the data to the average of all the electrodes.

ERPs for each child were averaged separately for each condition and baseline-corrected to the average voltage in the 100 ms prestimulus period.

As described above, EEG data collection was only possible for infants in Cohort 2 who participated in an in-person visit at 6 or 12 months (*n* = 116 in Cohort 2; *n* = 89 who completed an in-person 6-month visit; *n* = 94 who completed an in-person 12-month visit), following the lifting of pandemic-related research restrictions.

At 6 months of age, 78 children completed the EEG oddball task. EEG data were not collected from eleven infants due a technical error during EEG recording (*n* = 2) or because it was not possible to collect the data (*n* = 9; due to either child fussiness or parent refusal). Altogether, of the 78 participants who completed the MMR task, one participant’s data was excluded during preprocessing due to not having enough usable data (i.e., fewer than 20 artifact-free trials), leaving 77 participants with complete and usable MMR data at the 6-month time point.

At 12 months of age, 80 children completed the oddball task. EEG data were not collected from 12 infants due to the baby being fussy or the parent declining the measure. Altogether, of the 80 participants who completed the task at 12 months, 76 participants had complete and usable MMR data at the 12-month time point. Between the two time points at which EEG data was collected, 61 had data at both 6- and 12-month time points.

#### 2.2.8 | Mismatch Response

Based on visual inspection of the grand-averaged data and consistent with prior work examining associations between the MMR and language outcomes (Campos et al. 2023), neural responses to standards and deviants were quantified as the average activity across a frontal left cluster of channels (electrodes E20, E24, E28, E27, E34, E40) from 200 to 350 ms post-stimulus onset. We then computed the MMR by subtracting neural responses to standard stimuli from neural responses to deviant stimuli (deviant minus standard). To determine the minimum number of trials needed to obtain reliable estimates of the MMR, we examined Cronbach’s *α* as a function of an increasing number of deviant and standard trials.

The internal consistency reliability of the MMR at 6-months was low (*α* = 0.05) at 20 trials (the cutoff for usability in the current study). The internal consistency reliability of the MMR remained low (*α* < 0.50) at extended trial thresholds: 30 (*α* = 0.14), 40 (*α* = 0.10), 50 (*α* = 0.25), and 60 (*α* = 0.22) trials. Similarly, the internal consistency reliability of the MMR at 12 months was generally poor across different trial thresholds: *α* = 0.25 at 20 trials; 0.33 at 30 trials, 0.37 at 40 trials, 0.30 at 50 trials, and 0.39 at 60 trials. While low, these reliability values are similar to those observed in other child samples (Uwer and von Suchodoletz 2000; Martin et al. 2008). The number of participants dropped significantly after calculating Cronbach’s alpha for 60 trials (only 37 participants had more than 70 trials at 6 months; only 42 participants had more than 70 trials at 12 months). To confirm our results across usability thresholds, robustness checks were run at both time points using different trial cut-offs in analyses examining predictors of the MMR.

### 2.3 | Procedure

All protocols in this study were conducted in accordance with the ethical standards set by the Declaration of Helsinki. Informed consent was obtained from all participants, and procedures were approved by the Institutional Review Board of Teachers College, Columbia University. For this study, data were collected from mothers and infants at four time points: Prenatally, at 1, 6, and 12 months. At the prenatal visit, mothers either visited the research laboratory or research assistants visited their home to collect data which, pertinent to the current analyses, included mother demographic information and questions related to parent education and household income and composition. At the 1-month visit, which occurred either remotely or in-person, infant demographic information was reported. At the 6- and 12-month visit, mother-infant dyads either completed study activities remotely due to the COVID-19 pandemic (Cohort 1) or were invited to participate in an in-person lab visit, with an option for remote visit when necessary (Cohort 2). Families from both cohorts were invited to participate in the LENA activity for additional compensation at the 6-month visit. All families received instructions on how to complete the LENA activity and were provided with mailing materials to return the device back to the lab. The MMR was elicited in infants in Cohort 2 by recording EEG during a passive oddball task during the 6- and 12-month visit.

### 2.4 | Analytic Samples

Altogether, analytic samples varied depending on variables of interest within each model, with SES and LENA data collected across both cohorts, and EEG data collected only for infants in Cohort 2. Analyses examining SES and LENA outcomes had 115 observations when examining ITN as a predictor (Cohort 1 *n* = 41; Cohort 2 *n* = 74) and 122 observations when examining parent education as a predictor (Cohort 1 *n* = 46; Cohort 2 *n* = 76). When examining SES and the MMR at 6 months of age, 74 observations had both ITN and MMR, and 77 observations had both parent education and MMR. At 12 months of age, 74 observations had both ITN and MMR, and 76 had both parent education and the MMR. When examining LENA variables as a predictor of the MMR at 6 months of age, 59 participants had usable data. When examining LENA variables as a predictor of the MMR at 12 months of age, 57 participants had complete data.

### 2.5 | Statistical Plan

All analyses were conducted in SPSS (Version 29) and R Statistical Software. For descriptive purposes, we examined potential cohort differences in demographic variables. Next, we calculated Pearson’s *r* to examine bivariate correlations among study variables of interest (see Table 2). Multiple linear regressions were performed to examine our research questions. To test potential mediation effects, bias-corrected bootstrapping via the PROCESS macro was conducted, with a 95% confidence interval (Hayes 2013).

First, we examined whether prenatal family socioeconomic circumstances (family ITN and average parent education level, examined in separate models) predicted differences in the magnitude of the MMR at 6 and 12 months of age. Next, we examined whether prenatal family socioeconomic circumstances predicted characteristics of the home language environment (AWC and CTC) at 6-months of age. We then examined whether characteristics of the home language environment (examined in separate models) were associated with MMR magnitude at 6 and 12 months of age. Consistent with contemporary mediation frameworks, we did not require a statistically significant total effect of SES on the MMR to test mediation, as indirect pathways may exist even in the absence of a direct association (Hayes 2013). Accordingly, we tested whether either AWC or conversational turns mediated associations between SES variables (family ITN and parent education level) and MMR magnitude at either time point.

Next, we tested whether prenatal SES (examined in separate models) was associated with home noise levels, followed by examining whether home noise levels were associated with the magnitude of the MMR at 6 and 12 months of age. Finally, we examined whether average home noise levels mediated any associations between either SES variable (family income and parent education) and the magnitude of the MMR at either time point.

All analyses controlled for child age (in months) and sex (dummy coded: 1 = female; 0 = male). For analyses with the MMR as the outcome variable, child bilingual status (dummy coded: 0 = no exposure to a second language in the home; 1 = any exposure to a second language; see Measures section for additional information) was also used as a covariate. For analyses examining the home language environment and home noise levels, device recording time (in hours) was used as a covariate. Cohort number (dummy coded 0 = Cohort 1; 1 = Cohort 2) was also used as a covariate for analyses predicting the home language environment and home noise levels. Mediation analyses controlled for child age at the time of EEG visit, child sex, child bilingual status, and LENA recording time.

### 2.6 | Sensitivity Analyses

Robustness checks included repeated analyses in which we used non-winsorized family ITN as predictor, (see Table S1 for results), additionally controlled for family ITN, parent education, and child ethnicity (see Table S2 for results). We additionally ran robustness checks for analyses predicting the MMR in which we included a number of usable deviant trials after preprocessing as an additional covariate (see Table S3 for results). Finally, we reran models to examine predictors of the MMR, incrementally increasing the cutoff for usable data by 10 usable deviant trials at a time (see Table S4.1–4.5 for results).

## 3 | Results

### 3.1 | Descriptive Statistics

Table 1 shows the characteristics of the full sample. See Table S1 for characteristics for those with usable EEG data at either time point (*n* = 92). We examined potential sociodemographic differences between dyads in Cohort 1 (*n* = 93) and those in Cohort 2 (*n* = 116). Participants in Cohort 2 reported higher family income (M = $199,906, SD = $361,417; *t* (190) = 1.97; *p* = 0.05) and higher parent education levels (M = 15.5 years, SD = 3.27, *t* (207) = 2.45; *p* = 0.013) compared to Cohort 1 (mean family income = $115,304.65, SD = $168,677.53; mean parent education level = 14.3 SD = 3.31). In addition, there were more children identified as Hispanic/Latinx in Cohort 1 (*n* = 49) compared to children in Cohort 2 (*n* = 37) (*χ*^2^(2) = 10.61; *p* = 0.005). No cohort differences were found in regard to child race (*χ*^2^ (5) = 8.82; *p* = 0.12) or child bilingual status (*t* (184) = 1.10; *p* = 0.27).

### 3.2 | ERP Grand Average

**FIGURE 1:**
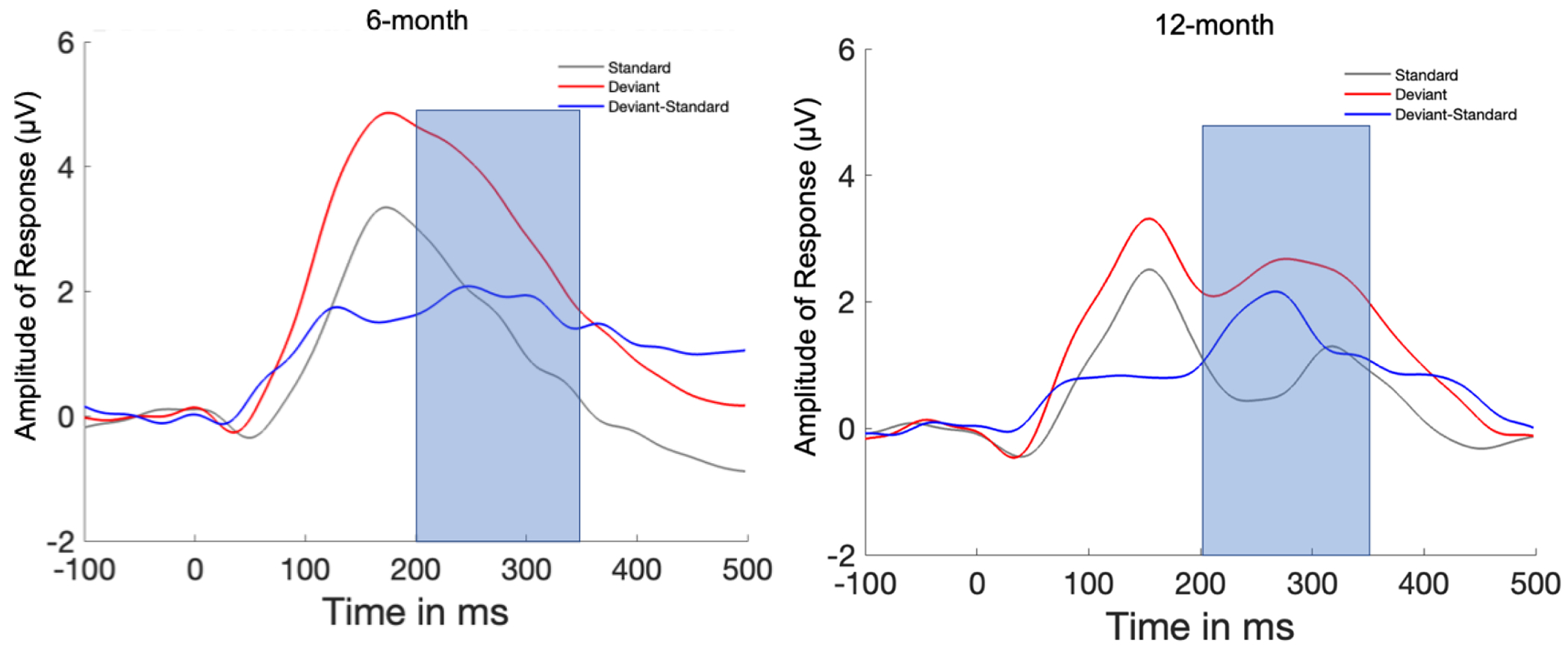
Grand Average ERPs. *Note*. ERPs were scored at an average of the frontal-left electrode cluster (channels E20, E24, E27, E28, E34, and E40) at 6- (left) and 12- (right) months of age. Highlighted area indicates ERP time window extracted for analysis (200-350 ms).

**FIGURE 2.**
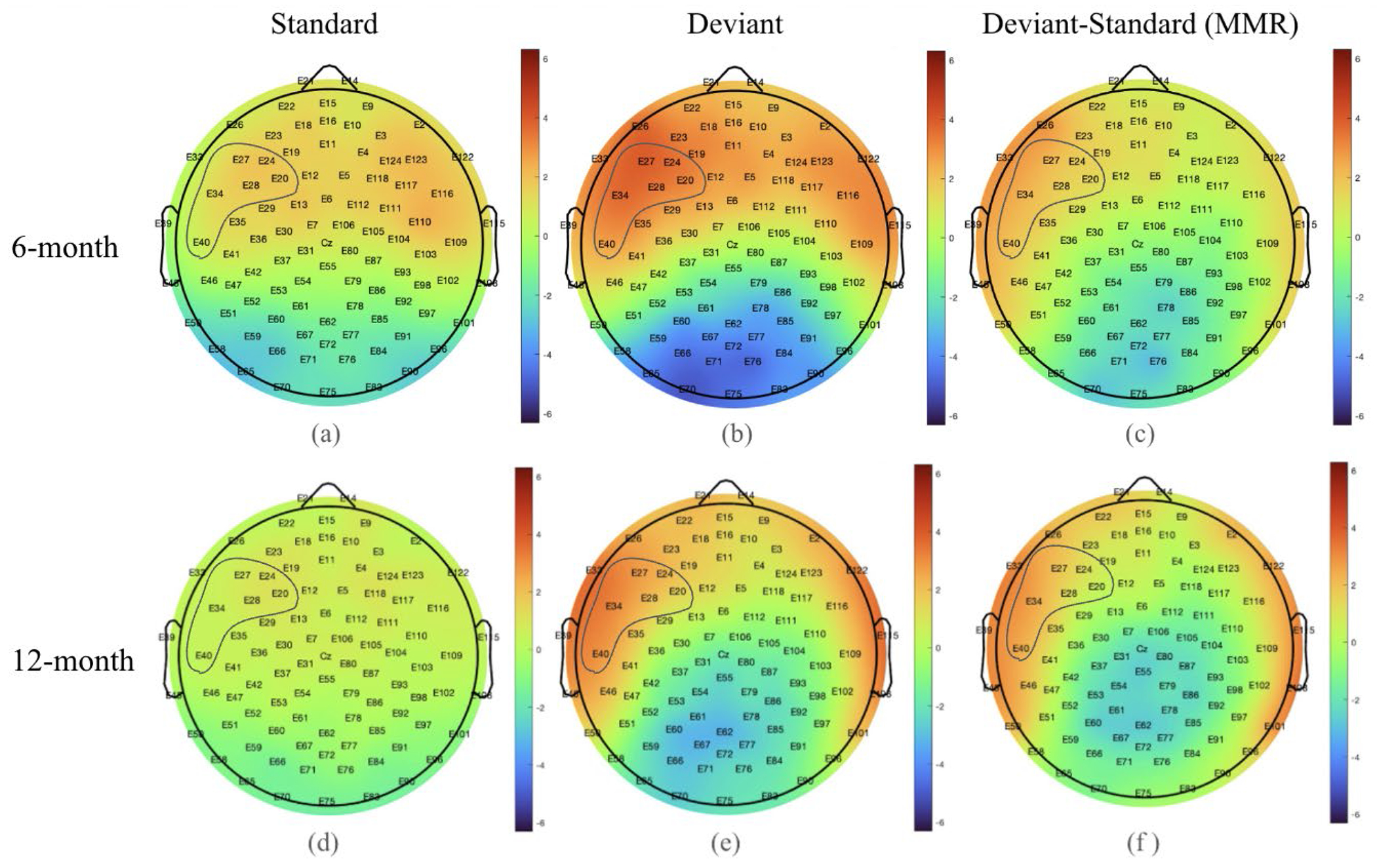
Topographic maps at 6 months (top) and 12 months (bottom). Topographic maps depict voltage across the scalp in the time window of the MMR (200 to 350 ms) for the standard(a,d), deviant(b,e), and difference between standard and deviant stimuli (c,f). Electrodes from which MMR amplitude was extracted are circled.

Figure 1 displays grand-average stimulus-locked ERPs in response to standard and deviant sounds, and the difference between them (MMR; deviant minus standard) at both time points. Topographic maps depicting voltage across the scalp for the standard response, deviant response, and MMR (deviant minus standard) are depicted in Figure 2. As depicted in Figure 1, the mean amplitude of the neural response to deviant stimuli at both time points was larger (i.e., more positive) than that of the standard stimuli (6-month time point: *t* (76) = 5.68, *p* < 0.001; 12-month time point: *t* (75) = 5.12, *p* < 0.001) during our time period of interest (200–350 ms post stimulus onset). The mean amplitude of the MMR did not differ by infant age at visit, sex at birth, race, or ethnicity at either time point (all *p*s > 0.05). At 12 months of age, mean amplitude was associated with child bilingual status in that bilingual children showed a reduced MMR magnitude *t* (70) = − 1.94, *p* = 0.05.

### 3.3 | Association Between Prenatal SES and the MMR Amplitude

Controlling for child age, sex, and child bilingual status, neither family ITN (*β* = 0.04; *p* = 0.77) nor parent education (*β* = −.05; *p* = 0.70) predicted MMR mean amplitude at 6-months of age. Similarly, neither family ITN (*β* = −0.003; *p* = 0.98) nor parent education (*β*= 0.06; *p* = 0.64) predicted MMR mean amplitude at 12 months of age.

### 3.4 | Association Between SES and Characteristics of the Home Language Environment

Controlling for child age, sex, device recording time, and cohort number, higher family ITN (*β* = 0.36 *p* < 0.001) and higher parent education (*β* = 0.25 *p* = 0.008) were associated with a higher average hourly AWC. Both higher family ITN (*β* = 0.33; *p* < 0.001) and higher parent education (*β* = 0.33; *p* < 0.001) were associated with higher average hourly CTC, replicating prior work in this sample (Hart et al. 2023). When running models without the infants whose LENA recordings had more than 3 pauses or spanned over 2 days (*n* = 5 dropped from analytic sample of those with LENA and SES data), results remained similar: Higher ITN and higher parent education predicted higher average hourly AWC (ITN: *β* = 0.36; *p* < 0.001; parent education: *β* = 0.25; *p* = 0.008), and higher average hourly CTC (ITN: *β* = 0.33, *p* < 0.001; parent education: *β* = 0.32; *p* < 0.001).

### 3.5 | Associations Between SES and Home Noise Levels

Controlling for child age, sex, conversational turns, cohort, and device recording time, neither family ITN (*β* = 0.06; *p* = 0.51) nor parent education levels (*β* = −0.10; *p* = 0.30) were significantly associated with home noise levels. When removing the 5 participants with LENA data containing more than three pauses or spanning more than 2 days, results remained similar; ITN: (*β* = 0.04; *p* = 0.71); parent education: (*β* = −0.08; *p* = 0.38).

### 3.6 | Associations Between Characteristics of the Home Language Environment and the MMR

Controlling for child age, sex, device recording time, and child bilingual status, neither hourly AWC (*β* = −0.03; *p* = 0.85) nor CTC (*β* = 0.14; *p* = 0.31) were associated with the magnitude of the MMR at 6 months of age. Results remained similar when excluding infants whose LENA recordings had more than three pauses or spanned over 2 days ( *n* = 1 dropped from analytic sample of those with LENA and MMR at 6-month time point).

Using the same covariates, neither CTC (*β* = 0.07; *p* = 0.64) nor hourly AWC were associated with the magnitude of the MMR at 12 months of age (*β* = −0.14; *p* = 0.31). Results remained similar when excluding participants without infants whose LENA recording had more than three pauses or spanned 2 or more days (*n* = 1 dropped from analytic sample).

### 3.7 | Associations Between Home Noise Levels and the Magnitude of the MMR

Home noise levels were not associated with the magnitude of the MMR at either time point, controlling for child age, sex, device recording time, bilingualism, and average hourly CTC, (6-month MMR: *β* = −0.10; *p* = 0.52; 12-month MMR: *β* = 0.06; *p* = 0.72). When running these models without the infants whose LENA recordings had more than three pauses or spanned over 2 days (*n* = 1 dropped from analytic sample), results remained similar.

### 3.8 | Mediation Results

Although SES was not directly associated with the MMR at either time point, mediation analyses can still be informative, as indirect effects may occur in the absence of a total effect (Hayes 2013, 2018, 2022). As such, we continued with our original statistical plan and examined potential indirect effects between SES and the MMR at both time points through the home language environment and home noise levels. Contrary to our hypotheses, no significant indirect effects were observed for either mediator. For the home language environment, the 95% bias-corrected bootstrapped confidence intervals for all indirect paths included zero (parent education and 6-month MMR: [95% CI: −0.01, 0.19]; family ITN and 6-month MMR: [95% CI: −0.03, 0.18]; parent education and 12-month MMR: [95% CI: −0.07, 0.10]; family ITN and 12-month MMR: [95% CI: −0.05, 0.11]). The same was true for home noise levels (parent education and 6-month MMR: [95% CI: −0.04, 0.04]; family ITN and 6-month MMR: [95% CI: −0.07, 0.07]; parent education and 12-month MMR: [95% CI: −0.05, 0.04]; family ITN and 12-month MMR: [95% CI: −0.08, 0.09]).

### 3.9 | Sensitivity Analyses

Results remained similar when using non-winsorized family ITN as a predictor (see Table S2), when controlling for supplemental covariates, including parent education and ITN, child ethnicity, and number of deviant trials (see Tables S3 and S4). Results also remained consistent when testing associations with increasing trial cutoffs (see Table S5.1–5.5)

## 4 | Discussion

The present study adds to a growing body of literature investigating factors that might predict brain function related to auditory discrimination in infancy. We examined whether socioeconomic factors (i.e., family ITN ratios and parent education), characteristics of the home language environment (i.e., AWC and adult-child conversational turns), and home noise levels might predict the magnitude of the MMR, an ERP commonly used to index auditory discrimination, at two time points in infancy. We further examined whether the home language environment or home noise levels would mediate associations between SES and the MMR at either time point. SES was not associated with the MMR at either time point. Similarly, neither metric of the home language environment (hourly CTC or AWC), nor home noise levels predicted MMR magnitude at either time point.

While this is among the first studies to look at SES, the home language environment, and home noise exposure in relation to the MMR within the same sample, prior work has examined different components of this research question. Although some work has found associations between SES and the MMR, directionality has been mixed. One study of infants aged 6–14 months found evidence that lower SES was associated with reduced amplitude of the N2, an ERP which can often overlap with the MMR and reflects the automatic detection of unexpected stimulus change (Wienke and Mathes 2024). Other work, however, has found evidence of more negative MMRs in adults who retroactively reported growing up in lower-SES households (Hao and Hu 2024). In contrast, we found no associations between SES and MMR amplitude in our sample. One potential explanation for these differences might be differences in how SES was conceptualized in each study. For example, Wienke and Mathes (2024) utilized parent education level (dichotomously rated as “low” or “high”) and migration background as their proxy for SES, while Hao and Hu (2024) used participant retrospective ratings on the MacArthur Scale of Subjective Social Status as a proxy for childhood SES. Our study, in contrast, used two continuous indices of SES: average parent education in years and family ITN. As such, it is possible that differences in results between studies are due to differences in how SES was measured. Future studies in which multiple dimensions of SES are measured from the same sample are warranted to best understand the potential associations between socioeconomic circumstances and the MMR.

Turning to the home language environment, we similarly did not find associations between home language environment and the MMR at either time point. This is in contrast to prior work in which language input predicted the MMR in older infants (Garcia-Sierra et al. 2016). In that study, bilingual 11- and 14-month-old infants who experienced high language input showed a larger, more positive MMR when listening to tones in their native language, indicative of stronger discrimination responses between stimuli (Garcia-Sierra et al. 2016). From these findings, we predicted that both AWC and CTC would be associated with the MMR. Notably, Garcia-Sierra and colleagues examined only the number of adult words during LENA recording segments reported to have the highest AWCs. In contrast, the current study examined both average AWC and CTC across the entire recording. These indices were selected to capture a more accurate estimate of the infant’s typical exposure to language across the entire day (excluding periods of sleep), rather than relying on brief periods of high language activity that may overestimate language input. Further, Garcia-Sierra and colleagues did not examine CTCs, which we included given previous work linking conversational turns with both language development and neural processing (Garcia-Sierra et al. 2016). It is possible that these analytic differences may explain discrepancies in findings across studies.

Characteristics of the home language environment have been associated with neural structure (Merz et al. 2020; Romeo et al. 2018) and function (Romeo et al.2018; Brito et al. 2020; Pierce et al. 2021) supporting language development, making language input a key predictor of early neurobiological mechanisms that support language. We hypothesized that both CTC and AWC would predict differences in MMR amplitude, with stronger associations for CTC, given that conversational turns require active engagement by the child. However, we saw no association between either index of the home language environment and MMR mean amplitude at either time point of interest. In contrast to the study by Garcia-Sierra and colleagues, in which bilingual and monolingual children were examined separately, the current study grouped bilingual and monolingual children together for analysis, utilizing binary labels of monolingual/bilingual as a covariate. It is important to consider bilingual status (i.e., bilingual, monolingual) when examining the MMR with linguistic stimuli, as the magnitude of the MMR has been shown to differ depending on whether stimuli are from native or non-native speech-sounds. Future work may also benefit by taking into account varying degrees of multilingualism when examining home language input and early indices of auditory discrimination.

Finally, we did not find evidence that noise levels in the home were associated with MMR amplitude. It is possible that such associations may not appear until later in childhood. It is also possible that other indices of neural function are instead affected by noise levels, such as brain structure, or other ERP components. Recent work has suggested that it may not be noise levels per se that are associated with language development, but rather speech-to-noise ratios (Benítez-Barrera et al. 2025). Prior studies examining brain function in the context of noisy environments have found reduced MMRs while in noisy conditions (Kozou et al. 2005). In contrast, our study elicited the MMR in quiet conditions, instead focusing on noise levels recorded in the home environment. It is possible that children with higher noise levels at home do not differ from those with lower noise levels at home under quiet conditions, but instead might show differences under noisy contexts (Muller-Gass et al. 2001). Future studies should incorporate the use of different noise-related metrics (e.g. speech-to-noise ratios) and examine the MMR under varying conditions to more fully capture how noise might shape language development and underlying brain function.

Taken together, discrepancies between the current null findings and prior reports linking SES, language input, or noise exposure to the MMR may also reflect differences in ERP methodology across studies. Prior studies examining the MMR have differed across several criteria: The characteristics of the auditory stimuli, the level of difference between the deviant and standard stimuli, and the age of the study sample, each of which may shape the observed associations. Differences across studies highlight that the absence of SES-, home language environment-, or noise-related effects on the MMR in the present study may not necessarily indicate the absence of such effects in infancy. Rather, discrepancies may stem from differences in how the MMR is measured, as well as from variations in stimulus characteristics that can influence characteristics of the ERP.

While among the first studies to examine predictors of the MMR at multiple time points in infancy, this study is not without limitations. Our ERP exhibited poor internal consistency reliability at both time points. While common when eliciting ERPs from young populations, variation in the MMR between trials may contribute to the inability to detect predictors of the MMR across participants. Unfortunately, few other studies examining the MMR in infancy and early childhood have reported on reliability metrics, rendering it difficult to compare the reliability of our data to other studies using similar paradigms. To address this issue, toolboxes such as the READIE toolbox have been developed to facilitate easy incorporation and reporting of internal consistency reliability statistics of EEG/ERP data (Xu et al. 2024). Incorporating these metrics is crucial for improving the field’s understanding of ERP measures, as it allows researchers to assess the stability and consistency of their findings across trials and participants. Without such reporting, it can be difficult to determine whether observed effects reflect true neural responses or are influenced by measurement variability. By adopting reliability reporting, future work can enhance comparability across datasets, improve replication efforts, and strengthen conclusions drawn from infant and child ERP research.

Further, our study was limited by a smaller sample than originally planned due to data collection restrictions related to the COVID-19 pandemic. As a result, the study may have been underpowered to observe the small to moderate effect sizes often observed when predicting neural measures broadly (Clayson et al. 2019). In addition, after restrictions were lifted, a new cohort of infants was recruited, with families in the second cohort notably reporting significantly higher income and parent education levels. Given that only infants in the second cohort contributed to the ERP data used in this study, demographic differences between cohorts possibly influenced our findings and limit their generalizability. Data collection on both cohorts is ongoing and data across both cohorts will examined together at later study time points (e.g., 24-months, 36-months), increasing statistical power and socioeconomic diversity of the sample. Finally, although we only analyzed the MMR as our neural outcome, the development of other ERP components such as the N2 or P2 may also be sensitive to SES- and experience-related differences in auditory processing (Kushnerenko et al. 2002; Stevens et al. 2009). Future work incorporating multiple ERPs will be important for clarifying how distinct aspects of auditory function develop in relation to SES, the home language environment, and home noise levels.

Despite these limitations, and although our hypotheses were not borne out, the present findings provide preliminary evidence and contribute to emerging longitudinal research examining associations among socioeconomic factors, the home language environment, home noise levels, and the MMR in a diverse sample of infants at two time points. Future studies should employ larger samples to ensure statistical power and to robustly examine how different aspects of the home language environment might shape early neural development. Continuing to examine these and related factors over time will be critical to advancing our understanding of how early experiences influence brain and language development.

## Supplementary Material

Supplement

Additional supporting information can be found online in the Supporting Information section.

Table S1. Descriptive of children with EEG outcomes at either 6- or 12-months (*n* = 92) Table S2. Associations when using non-winsorized family income-to-needs (ITN) values as a predictor. S3. Predicting the MMR when controlling for additional covariates: ITN, ethnicity and parent education. S4. Associations when additionally controlling for number of usable trials. S5. Associations with increasing trial cutoffs.

## Figures and Tables

**TABLE 1 |:** Full sample characteristics.

		M	SD	Range		Minimum	Maximum

Infant age (in months) at 6-month EEG visit		6.78	0.95	5.23		5.23	10.47
Infant age (in months) at 12-month EEG visit		12.6	0.94	5.03		11.28	16.31
Infant age (in months) at LENA completion		7.46	1.16	5.67		5.40	11.07
Parental education (years)		15.00	3.33	16.00		6.00	22.00
Family income-to-needs ratio		6.85	8.84	43.74		0	43.74
Family income (in USD)		163,334	296,379	2,563,500		0	
							2,563,500.00
Number of standard trials: 6 months		425	99	405		155	566
Number of deviant trials: 6 months		74	18	77		23	100
MMR mean amplitude: 6 months (μV)		1.81	2.74	15.06		−5.78	9.28
Number of standard trials: 12 months		428	118	442		124	566
Number of deviant Trials: 12 months		75	21	75		25	100
MMR mean amplitude: 12 months (μV)		1.59	2.64	13.05		−5.23	7.83
		%			*n*		
Infant sex	Male	41.1%			86		
	Female	51.2%			107		
	Prefer not to answer	0.5%			1		
	Missing/Unknown	7.2%			15		
**Infant ethnicity**	Hispanic or Latino	41.1%			86		
	Not Hispanic or Latino	48.3%			101		
	Prefer not to answer	2.4%			5		
	Missing/Unknown	8.1%			17		
**Infant race**	White	34.9%			73		
	Black or African American	22.0%			46		
	Asian	3.8%			8		
	American Indian/Alaska Native	1.0%			2		
	Other	23.9%			50		
	Prefer not to answer	6.2%			13		
	Missing/Unknown	8.1%			17		
**Bilingual status**	Monolingual household	64.1%			134		
	Bilingual household	24.9%			52		
	Unknown/Missing	11.0%			23		

**TABLE 2 |:** Correlation table

	1	2	3	4	5	6	7	8	9	10	11	12
1. Mismatch response (MMR), 6 months	—											
2. Mismatch response (MMR), 12 months	0.03	—										
3. Family Income-to-Needs (ln)	0.02	0.00	—									
4. Parent education (years)	−0.04	0.07	0.54**	—								
5. Hourly adult word count	0.03	−0.13	0.35**	0.28**	—							
6. Hourly conversational turn count	0.18	0.04	0.31**	0.32**	0.51**	—						
7. Hourly child vocalization count	0.20	0.05	0.13	0.14	−0.04	0.66**	—					
8. Average noise level (dB)	−0.12	0.11	0.05	−0.10	0.02	0.20**	0.34**	—				
9. LENA recording time (hours)	−0.30*	0.07	−0.27**	−0.30	−0.13	−0.17	−0.10	0.34**	—			
10. Infant age when first MMR collected (months)	−0.11	−0.05	0.16*	0.01	−0.08	−0.07	0.09	0.01	−0.01	—		
11. Infant age when second MMR collected (months)	−0.02	−0.08	0.05	−0.08	−0.05	−0.00	−0.01	0.00	0.01	0.52**	—	
12. Infant age when LENA collected (months)	0.08	−0.15	0.10	−0.04	−0.16	−0.04	0.08	0.04	0.08	0.55**	0.23*	—

* Correlation is significant at the 0.05 level.

** Correlation is significant at the 0.01 level.

## Data Availability

The data that support the findings of this study are available from the corresponding author, KGN, upon reasonable request.
