# Supplementary material for: Socioeconomic Status, the Home Language Environment, Noise Exposure, and the Mismatch Response in Infancy
Source: Dev Psychobiol. Author manuscript; Available in PMC 2026 Jun 29. (PMC13005931; doi:10.1002/dev.70128)
Supplement: Supplement [file NIHMS2145754-supplement-Supplement.docx]

**Supplemental Materials**

**Table S1.** Descriptive of children with EEG outcomes at either 6- or 12-months (n=92)

|  | **M** | **SD** | **Range** |  | **Minimum** | **Maximum** |
| --- | --- | --- | --- | --- | --- | --- |
| **Infant age (in months) at LENA completion** | 7.10 | 1.02 | 5.36 |  | 5.32 | **10.68** |
| **Parental education (years)** | 15.45 | 3.16 | 16.00 |  | 6.00 | **22.00** |
| **Family income-to-needs ratio** | 6.85 | 8.84 | 43.74 |  | 0 | **43.74** |
| **Family income (in USD)** | 214,880 | 395,589 | 2,563,500 |  | 0 | **2,563,500** |

|  | | **%** |  |  | **n** |  |  |
| --- | --- | --- | --- | --- | --- | --- | --- |
| **Infant Sex** | Male | 45.7 |  |  | 42 |  |  |
|  | Female | 52.2 |  |  | 48 |  |  |
|  | Missing/Unknown | 2.2 |  |  | 2 |  |  |
| **Infant Ethnicity** | Hispanic or Latino | 34.8 |  |  | 32 |  |  |
|  | Not Hispanic or Latino | 59.8 |  |  | 55 |  |  |
|  | Prefer not to answer | 3.3 |  |  | 3 |  |  |
|  | Missing/Unknown | 2.2 |  |  | 2 |  |  |
| **Infant Race** | White | 41.3 |  |  | 38 |  |  |
|  | Black or African American | 28.3 |  |  | 26 |  |  |
|  | Asian | 4.3 |  |  | 4 |  |  |
|  | American Indian/Alaska Native | 0 |  |  | 0 |  |  |
|  | Other | 16.3 |  |  | 15 |  |  |
|  | Prefer not to answer | 7.6 |  |  | 7 |  |  |
|  | Missing/Unknown | 2.2 |  |  | 2 |  |  |
| **Bilingual Status** | Monolingual household | 73.9% |  |  | 68 |  |  |
|  | Bilingual household | 22.8% |  |  | 21 |  |  |
|  | Unknown/Missing | 3.3% |  |  | 3 |  |  |

**Table S2.** Associations when using non-winsorized family income-to-needs (ITN) values as a predictor.

As stated in the main text, three ITN values were detected as outliers and were winsorized prior to analysis. As such, results were run with and without winsorized ITN values, with results using winsorized values described in the main text. Results using non-winsorized ITN values are described below. All other covariates remained the same.

**Summary of standardized beta and significance levels for supplemental analyses examining non-winsorized ITN values as predictor variable of interest.**

| **Outcome Variable:** | **β** | **p-value** |
| --- | --- | --- |
|  |  |  |
| **Home Language Environment** |  |  |
| Adult World Count | .35 | <.001 |
| Conversational Turn Count | .30 | .002 |
| **Home Noise Exposure**  **MMR Magnitude (6-months)**  **MMR Magnitude (12-months)** | .05  .04  .04 | .57  .99  .73 |

Similar to results in the main analyses, ITN predicted both adult word count and conversational turn count. ITN did not predict home noise exposure nor MMR magnitude.

**S3. Predicting the MMR when controlling for additional covariates: ITN, ethnicity and parent education.**

As stated in the main text, cohorts differed by ethnicity, family income, and parent education levels. As such, we reran analyses predicting the MMR (only collected in cohort 2) at both timepoints with ethnicity, family income, and parent education as covariates (both SES variables were run in the same model, with ethnicity added as a covariate). In analyses in which both cohorts had data collected, cohort was already included as a covariate in the model, and as such was not included as a covariate in this set of robustness checks. All other covariates remained the same for each model (see note).

**Table S3.** Summary of standardized betas in supplemental analyses examining associations between 1) SES, 2) the home language environment, and 3) home noise levels, and MMR magnitude at 6- and 12-months when additionally controlling for ethnicity, ITN, and parent education.

|  |  |  | | **6-month MMR** | | | **12-month MMR** | |
| --- | --- | --- | --- | --- | --- | --- | --- | --- |
| **Predictor Variable** | | | **β** | | ***p*-value** | **β** | | ***p*-value** |
| **SES** | | |  | |  |  | |  |
| ITN | | | .12 | | .39 | .09 | | .56 |
| Parent Education | | | -.01 | | .94 | -.01 | | .94 |
| **Home Language Environment** | | |  | |  |  | |  |
| Adult World Count | | | -.01 | | .93 | -.17 | | .25 |
| Conversational Turn Count | | | .16 | | .23 | .06 | | .69 |
| **Home Noise Exposure** | | | -.14 | | .34 | -.08 | | .63 |

*Note.* All models were run separately and also controlled for covariates mentioned in the main text (i.e., child age, gender, bilingual status for all models, device recording time for the home language environment and home noise exposure, and hourly conversational turn count for home noise exposure).

**S4. Associations when additionally controlling for number of usable trials.**

As a robustness check, we reran analyses predicting the mismatch response (MMR) at both timepoints additionally controlling for number of usable trials, results generally remained similar.

**Table S4.** Summary of standardized betas in supplemental analyses examining associations between 1) SES, 2) the home language environment, and 3) home noise levels, and MMR at both timepoints when additionally controlling for number of deviant trials.

|  |  |  | **6-month MMR** | | | | **12-month MMR** | |
| --- | --- | --- | --- | --- | --- | --- | --- | --- |
| **Predictor Variable** | | | | **β** | ***p*-value** | **β** | | ***p*-value** |
| **SES** | | | |  |  |  | |  |
| ITN | | | | -.06 | .68 | .01 | | .94 |
| Parent Education | | | | -.16 | .25 | .07 | | .62 |
| **Home Language Environment** | | | |  |  |  | |  |
| Adult World Count | | | | -.03 | .81 | -.16 | | .24 |
| Conversational Turn Count | | | | .02 | .89 | .02 | | .87 |
| **Home Noise Exposure** | | | | -.09 | .54 | .08 | | .63 |

*Note.* Each predictor listed was run in a separate regression model with all other covariates indicated in the main text (i.e., child age, gender, and bilingual status for all models, plus total recording time for the home language environment and home noise variables, plus conversational turn count for home noise exposure).

**S5. Associations with increasing trial cutoffs.**

Given the relatively low reliability scores for the MMR at both timepoints (determined using Cronbach’s alpha, see main text), we also tested associations using different trial cutoffs as a robustness check. All covariates remained the same in all analyses.

**Table S5.1** Summary of standardized betas in supplemental analyses examining associations between 1) SES, 2) the home language environment, and 3) home noise levels, and MMR using a cutoff of 30 usable deviant trials (n=2 MMR values dropped at 6-months; n = 3 MMR values dropped at 12-months)

|  |  |  | **6-month MMR** | | | | **12-month MMR** | |
| --- | --- | --- | --- | --- | --- | --- | --- | --- |
| **Predictor Variable** | | | | **β** | ***p*-value** | **β** | | ***p*-value** |
| **SES** | | | |  |  |  | |  |
| ITN | | | | .037 | .77 | .04 | | .78 |
| Parent Education | | | | -.08 | .52 | .03 | | .81 |
| **Home Language Environment** | | | |  |  |  | |  |
| Adult World Count | | | | -.04 | .77 | -.14 | | .34 |
| Conversational Turn Count | | | | .13 | .33 | .06 | | .66 |
| **Home Noise Exposure** | | | | -.10 | .56 | .02 | | .90 |

Note: Each predictor listed was run in a separate regression model with all other covariates indicated in the main text (i.e., child age, gender and bilingual status for all models, plus total recording time for the home language environment and home noise variables, plus conversational turn count for home noise exposure).

**Table S5.2** Summary of standardized betas in supplemental analyses examining associations between 1) SES, 2) the home language environment, and 3) home noise levels, and MMR using a cutoff of 40 usable deviant trials (n=6 MMR values dropped at 6-months; n = 8 MMR values dropped at 12-months)

|  |  |  | **6-month MMR** | | | | **12-month MMR** | |
| --- | --- | --- | --- | --- | --- | --- | --- | --- |
| **Predictor Variable** | | | | **β** | ***p*-value** | **β** | | ***p*-value** |
| **SES** | | | |  |  |  | |  |
| ITN | | | | .06 | .64 | .02 | | .86 |
| Parent Education | | | | -.07 | .60 | -.03 | | .80 |
| **Home Language Environment** | | | |  |  |  | |  |
| Adult World Count | | | | .14 | .35 | -.135 | | .38 |
| Conversational Turn Count | | | | .12 | .42 | .01 | | .94 |
| **Home Noise Exposure** | | | | -.11 | .52 | .003 | | .98 |

*Note:* Each predictor listed was run in a separate regression model with all other covariates indicated in the main text (i.e., child age, gender, and bilingual status for all models, plus total recording time for the home language environment and home noise variables, plus conversational turn count for home noise exposure)

**Table S5.3** Summary of standardized betas in supplemental analyses examining associations between 1) SES, 2) the home language environment, and 3) home noise levels, and MMR using a cutoff of 50 usable deviant trials (n=9 MMR values dropped at 6-months; n = 11 MMR values dropped at 12-months)

|  |  |  | **6-month MMR** | | | | **12-month MMR** | |
| --- | --- | --- | --- | --- | --- | --- | --- | --- |
| **Predictor Variable** | | | | **β** | ***p*-value** | **β** | | ***p*-value** |
| **SES** | | | |  |  |  | |  |
| ITN | | | | .08 | .56 | .015 | | .91 |
| Parent Education | | | | -.07 | .57 | -.04 | | .77 |
| **Home Language Environment** | | | |  |  |  | |  |
| Adult World Count | | | | .22 | .15 | -.13 | | .39 |
| Conversational Turn Count | | | | .12 | .42 | -.01 | | .96 |
| **Home Noise Exposure** | | | | -.28 | .12 | -.01 | | .94 |

*Note:* Each predictor listed was run in a separate regression model with all other covariates indicated in the main text (I.e., child age, gender, and bilingual status for all models, plus total recording time for the home language environment and home noise variables, plus conversational turn count for home noise exposure)

**Table S5.4.** Summary of standardized betas in supplemental analyses examining associations between 1) SES, 2) the home language environment, and 3) home noise levels, and MMR using a cutoff of 60 usable deviant trials (n=12 MMR values dropped at 6-months; n = 16 MMR values dropped at 12-months)

|  |  |  | **6-month MMR** | | | | **12-month MMR** | |
| --- | --- | --- | --- | --- | --- | --- | --- | --- |
| **Predictor Variable** | | | | **β** | ***p*-value** | **β** | | ***p*-value** |
| **SES** | | | |  |  |  | |  |
| ITN | | | | .16 | .22 | -.06 | | .67 |
| Parent Education | | | | -.06 | .64 | -.04 | | .79 |
| **Home Language Environment** | | | |  |  |  | |  |
| Adult World Count | | | | .23 | .15 | -.11 | | .52 |
| Conversational Turn Count | | | | .12 | .42 | .008 | | .96 |
| **Home Noise Exposure** | | | | -.27 | .15 | -.02 | | .90 |

*Note:* Each predictor listed was run in a separate regression model with all other covariates indicated in the main text (I.e., child age, gender, and bilingual status for all models, plus total recording time for the home language environment and home noise variables, plus conversational turn count for home noise exposure)

**Table S5.5.** Summary of standardized betas in supplemental analyses examining associations between 1) SES, 2) the home language environment, and 3) home noise levels, and MMR using a cutoff of 70 usable deviant trials (n=20 MMR values dropped at 6-months; n = 26 MMR values dropped at 12-months).

|  |  |  | **6-month MMR** | | | | **12-month MMR** | |
| --- | --- | --- | --- | --- | --- | --- | --- | --- |
| **Predictor Variable** | | | | **β** | ***p*-value** | **β** | | ***p*-value** |
| **SES** | | | |  |  |  | |  |
| ITN | | | | .10 | .48 | -.25 | | .07 |
| Parent Education | | | | -.20 | .16 | -.17 | | .26 |
| **Home Language Environment** | | | |  |  |  | |  |
| Adult World Count | | | | .27 | .11 | -.09 | | .59 |
| Conversational Turn Count | | | | .14 | .40 | -.11 | | .48 |
| **Home Noise Exposure** | | | | -.26 | .18 | -.15 | | .43 |

*Note:* Each predictor listed was run in a separate regression model with all other covariates indicated in the main text (i.e., child age, gender, and bilingual status for all models, plus total recording time for the home language environment and home noise variables, plus conversational turn count for home noise exposure)
